# Massachusetts Dental Society

**Published:** 1866-07

**Authors:** 


### MASSACHUSETTS DENTAL SOCIETY.

We clip the following from a Boston paper.—Ed.

The annual meeting of the Massachusetts Dental Society was held at theSuffolk District Medical Society's rooms, in Perkins Building, Temple Place, yesterday, at 10 o'clock. In the absence of the President, the meeting was called to order by Dr. E. G. Leach. About fifty members were present from different parts of the State, and the forenoon was devoted to a variety of clinical operations upon different subjects, performed by N. C. Keep, M. D., of Boston, President of the Society; H. F. Bishop, D. D. S., of Worcester; I. J. Weatherbee, D. D. S., of Boston; L. D. Shepard, D. D. S., of Salem; Drs. E. G. Leach, and I. A. Salmon of Boston.

The afternoon was devoted to the election of officers for the ensuing year, reports of committees, &c.

The election of officers of the Society for the ensuing year resulted in the choice of the following: President, N. C. Keep M. D., Boston; Vice-President, Dr. E. G. Leach, Boston; Recording Secretary, L. D. Shepard, D. D. S.., Salem; Corresponding Secretary, E. C. Rolfe, M. D., Boston; Treasurer, S. G. McDougall, M. D., Boston; Librarian, E. N. Harris, D. D. S.; Executive Committee, I. J. Weatherbee, D. D. S., Boston, Dr. J. Y. Codman, Boston; Dr. Geo. T. Moffat, Boston, Dr. E. Blake, Stoughton, H. F. Bishop, D. D. S., Worcester.

H. F. Bishop, D. D. S. of Worcester, was elected to deliver the next annual address. A. Lawrence, D. D. S. of Lowell was elected substitute.

The Society then listened to the delivery of the annual address by Isaac J. Weatherbee, D. D. S. of Boston, which gave an extended and interesting history of the formation of the Society, its growth, objects and success, urging upon its members the importance of improving every opportunity of perfecting the science and of imparting to all who come under their treatment the necessity for their general health, of a proper care of their teeth.

After the delivery of this address, which was listened to with great interest and frequently applauded, a committee to make arrangments on the part of the Society for the proper reception of the American Dental Association, which is to convene in this city July 31, was appointed, consisting of the following: Drs. N. C. Keep, I. J. Weatherbee, E. G. Leach, T. H. Hitchcock, I. A. Salmon, E. C. Rolfe, B. S. Codman, L. D Shepard and A. Lawrence.

The following were chosen delegates to the American Dental Association: Drs. Keep, Leach, E. N. Harris, T. H. Chandler, Hitchcock, Rolfe, McDougal, Moffat and Dotty of Boston; W. L. Bowdoin of Salem, O. F. Harris of Worcester and A. A. Cook of Milford.

The reports of the different officers of the Society show it in a flourishing condition in every respect. A number of valuable additions, by donation and otherwise, have been made to the library and cabinet, and their financial condition is very satisfactory.

At the close of the general business of their meeting the members of the society, together with a number of invited guests, repaired to the Tremont House, to partake of the annual dinner served in the enticing style for which the house is noted.

After due attention to the substantials and delicacies of the table, the repast of a higher order was commenced and continued until nearly seven o'clock, consisting of very happy after dinner speeches from the President, Dr. Keep, Drs. A. A. Gould, Horn, Weatherbee, Leach, L. D. Shepard, of Salem, T. H. Hitchcock, Calvin Page, Bowditch, Palmer and others. During the speeches of the dinner and discussions, while in the transaction of the business at their rooms, the attention of the society was frequently called by members of the dental and medical profession to the demand existing for the establishing of some permanent place where the two professions could meet frequently for mutual interchange of information, and where could be kept the publications on dentistry and medicine that are constantly being issued, to be accessible to physicians and dentists, and the hope expressed that the time was not far distant when New England would have a dental college, or that the professorship of dentistry would be soon added to the Boston Medical School.

After the transaction of unimportant matters of business, a discussion upon the best methods and means to be employed in filling teeth was engaged in very generally by the members of the society, at the close of which the meeting adjourned.

